# Supplementary material for: Cryopreservation Cooling Rate Impacts Post-Thaw Sperm Motility and Survival in Litoria booroolongensis
Source: Animals (Basel). 2023 Sep 25;13(19):3014. doi: 10.3390/ani13193014 (PMC10571529; doi:10.3390/ani13193014)
Supplement: Supplementary file 1 [file animals-13-03014-s001.zip › animals-2560616-supplementary.pdf]

Supplementary Table S1. Estimated marginal means (EMMs) and 95% confidence intervals (LCL, UCL) for total motility and forward progressive motility at time 0 and 30-minutes post-thaw.

			Total Motility			Forward progressive motility		
Cryoprotectant	Cooling Rate	Assessment Time (min. post-thaw)	EMM	LCL	UCL	EMM	LCL	UCL
CPAA	FrA	0	0.361	0.263	0.472	0.0676	0.0414	0.108
		30	0.309	0.220	0.415	0.0623	0.0380	0.101
	FrB	0	0.621	0.509	0.722	0.1154	0.0730	0.178
		30	0.565	0.451	0.673	0.1069	0.0671	0.166
CPAB	FrA	0	0.444	0.335	0.559	0.0766	0.0472	0.122
		30	0.388	0.285	0.501	0.0707	0.0433	0.113
	FrB	0	0.589	0.477	0.692	0.1299	0.0831	0.197
		30	0.532	0.419	0.641	0.1204	0.0765	0.185
